# Route of antigen delivery impacts the immunostimulatory activity of dendritic cell-based vaccines for hepatocellular carcinoma

**DOI:** 10.1186/s40425-015-0077-x

**Published:** 2015-07-21

**Authors:** Angela D. Pardee, Hiroshi Yano, Aliyah M. Weinstein, Aaron A. K. Ponce, Alexander D. Ethridge, Daniel P. Normolle, Lazar Vujanovic, Gerald J. Mizejewski, Simon C. Watkins, Lisa H. Butterfield

**Affiliations:** Departments of Medicine, Pittsburgh, PA 15261 USA; Departments of Biostatistics, Pittsburgh, PA 15261 USA; Departments of Surgery, Pittsburgh, PA 15261 USA; Departments of Immunology, University of Pittsburgh School of Medicine, Pittsburgh, PA 15261 USA; University of Pittsburgh Cancer Institute, Hillman Cancer Center 5117 Centre Avenue, Suite 1.27, Pittsburgh, PA 15213 USA; Non-paid Advisor at the Wadsworth Center, New York State Department of Health, Albany, NY 12201 USA; Department of Cell Biology and Physiology, University of Pittsburgh, Pittsburgh, PA 15261 USA

**Keywords:** Alpha-fetoprotein, Dendritic cells, Hepatocellular carcinoma, Adenovirus

## Abstract

**Background:**

Dendritic cells (DC) are uniquely equipped to capture, process, and present antigens from their environment. The context in which an antigen is acquired by DC helps to dictate the subsequent immune response. Cancer vaccination promotes antitumor immunity by directing an immune response to antigens expressed by tumors. We have tested the tumor-associated antigen alpha-fetoprotein (AFP) as an immunotherapy target. The majority of hepatocellular carcinomas (HCC) upregulate and secrete this oncofetal antigen.

**Methods:**

To develop cancer vaccines for HCC capable of promoting potent tumor-specific T cell responses, we tested adenovirally-encoded synthetic AFP, with or without its signal sequence, as well as protein forms of AFP and compared intracellular routing and subsequent antigen-specific CD8+ and CD4+ T cell responses.

**Results:**

Surprisingly, the secreted form of antigen was superior for both CD4+ and CD8+ T cell activation. We also examined the mechanism through which AFP protein is endocytosed and trafficked in human DC. We identify the mannose receptor (MR/CD206) as the primary uptake pathway for both normal cord blood-derived AFP (nAFP) and tumor-derived AFP (tAFP) proteins. While in healthy donors, nAFP and tAFP were cross-presented to CD8+ T cells similarly and CD4+ T cell responses were dependent upon MR-mediated uptake. In HCC patient cells, tAFP was more immunogenic, and CD4+ T cell responses were not MR-dependent.

**Conclusions:**

Secreted, cytoplasmically retained, and endocytosed forms of AFP utilize unique uptake and processing pathways, resulting in different immunologic responses from the induced antigen-specific CD4+ and CD8+ T cells and between healthy donors and HCC patients. Collectively, these data elucidate pathways of spontaneous and induced anti-tumor immunity in HCC patients to this secreted antigen.

**Electronic supplementary material:**

The online version of this article (doi:10.1186/s40425-015-0077-x) contains supplementary material, which is available to authorized users.

## Background

DC play a key role in initiating the adaptive immune response by sampling antigens from their environment and presenting them to lymphocytes. The physiologic cues received by DC as antigen is taken up impact processing and presentation and help to shape subsequent lymphocyte responses [1, 2]. DC were shown initially to utilize both (macro)pinocytosis and the mannose receptor (MR/CD206), a C-type lectin receptor (CLR), to take up and concentrate model antigens like horseradish peroxidase and mannosylated bovine serum albumin (BSA) [3]. Scavenger receptors, CLRs, heat shock proteins, and Fc receptors are also antigen uptake mechanisms used by DC [4]. Endocytosis of the model protein antigen ovalbumin via scavenger receptors promotes lysosomal degradation of ovalbumin, MHC II-restricted presentation, and activation of CD4+ T cells. In contrast, uptake via MR results in enhanced cross-presentation of antigen to CD8+ T cells [5], which was recently shown to involve Sec61 mediated transport between endosomes and the cytosol [6]. Additional studies suggest that antigen uptake by receptors that facilitate early endosomal routing and accumulation of antigen in endosomes, rather than lysosomal delivery and proteolytic digestion, should promote cross-presentation, prolonged antigen presentation and subsequently induce superior CD8+ T cell activation, an essential component of anti-tumor immunity [7, 8].

Alpha-fetoprotein (AFP) is the most abundant serum protein in the fetus, reaching levels of up to 3 mg/ml in fetal blood [9]. It is transcriptionally repressed shortly after birth, and normal adult levels are between 1–20 ng/ml. The reappearance of AFP in the circulation of adults is associated with liver regeneration, hepatitis, chronic liver diseases and malignant growth, including hepatomas and teratomas [10]. Serum assays of circulating AFP play an important role in the diagnosis of hepatocellular carcinoma (HCC) and monitoring responses to treatment. In contrast to fetal AFP, tumor-secreted AFP is differentially glycosylated [11, 12]. A fucosylated variant, known as AFP-L3, is the major glycoform in HCC patient serum and is associated with poor outcome [13]. The uptake of AFP by multiple cell types, including epithelial cells, activated lymphocytes and tumor cells, has been observed [14–17], although a specific cell surface receptor has not yet been definitively identified [17].

Given its common over-expression by the majority of HCC tumors, AFP represents an attractive target for immunotherapy [18]. It has been demonstrated that both CD4+ and CD8+ T cells can recognize AFP epitopes presented by dendritic cells (DC) [19]. Our group observed that CD8+ T cells from healthy donors can respond to four dominant and ten subdominant HLA-A^*^0201-restricted peptides *in vitro* [20]. At least three clinical trials have tested AFP-based vaccine regimens: i) four immunodominant HLA-A*0201-restricted AFP peptides emulsified in Montanide adjuvant [21], ii) AFP peptide-pulsed autologous DC [22], and iii) a DNA-prime/adenovirus (AdV)-boost genetic immunization [23]. Although no objective clinical responses were observed in the small numbers of vaccinated patients, AFP-specific T cell responses were either developed or expanded in the majority of patients. The association between AFP secretion and poor clinical outcome, HCC stemness [24] and tumor growth rate supports further testing of AFP as an immunogenic tumor-associated antigen target. Because of the inherent variability in human self-tumor antigen responses and the small size of most cancer vaccine clinical trials, it is not yet clear how to load DC with antigen optimally for CTL induction. Clinical trials continue to utilize a wide array of antigen sources and uptake pathways to attempt to promote antitumor immunity. It is also increasingly clear that there is considerable tumor-immune crosstalk before tumors become clinically evident, and many patients have spontaneous immune responses to tumor antigens without vaccination or other therapy.

In this study, we examined different forms of AFP antigen to identify how the antigen is taken up, processed, and presented by DC. By investigating the fetal and tumor-induced immunity to this secreted antigen and examining the subsequent impact on T cell responses, we inform the design of future vaccination strategies targeting this oncofetal antigen.

## Results and discussion

### AdV-transduction induces partial maturation of DC

**Fig. 1:**
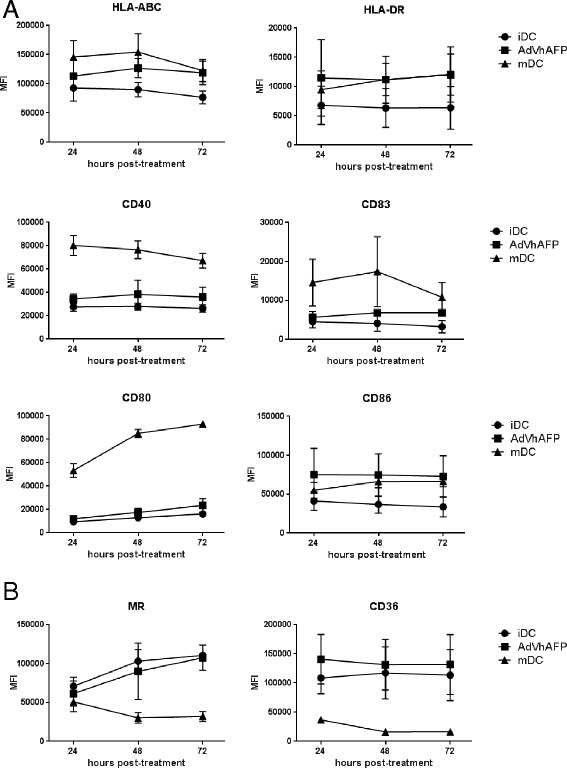
Phenotype of AdV-transduced DC. **a** and (**b**) Immature DC (iDC) from healthy donors (n = 3) were left untreated, matured with LPS/IFN-γ (mDC), or transduced with AdVhAFP, and then cultured in DC media for 24, 48, or 72 hr. Cells were stained for (**a**) antigen presentation and costimulatory markers and (**b**) endocytic receptors, and analyzed by flow cytometry. Mean fluorescence intensity (MFI) is reported as the mean ± SD

We have previously utilized adenoviral vectors for genetic engineering of DC due to their ability to express full length antigens within DC and positively impact some aspects of DC function [25–29]. To further characterize the maturation effects of AdV on DC, we first transduced healthy donor (HD) DC with an AFP-encoding AdV (AdVhAFP) and monitored the expression of several maturation markers over the course of 3 days. Compared to immature DC (iDC) and LPS/IFN-γ-matured DC (mDC), AdV-transduced DC exhibited intermediate expression levels of antigen presentation molecules (HLA-ABC, HLA-DR) and costimulatory molecules (CD40, CD83, CD80, CD86) (Fig. 1a). We also analyzed expression of the endocytic receptors MR and CD36 following AdV-transduction (Fig. 1b). Unlike mDC, which highly downregulate these receptors, AdV-transduced DC express levels similar to iDC, suggesting that AdV infection does not compromise the endocytic function of DC.

### Adenovirally-expressed AFP localizes to the Golgi apparatus and related compartments in DC

**Fig. 2:**
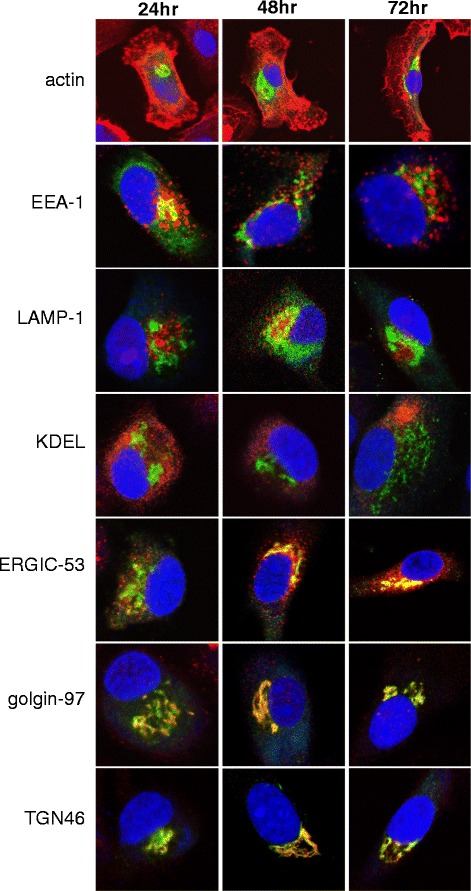
Intracellular trafficking of adenovirally-expressed AFP. DC were transduced with AdVhAFP at MOI 2000 for 3 hr, and then cultured in DC media for 24, 48, or 72 hr. Cells were then fixed and stained for AFP (green), actin, EEA-1 (early endosomes), LAMP-1 (late endosomes/lysosomes), KDEL (endoplasmic reticulum/ER), ERGIC-53 (ER-Golgi intermediate complex), golgin-97 (Golgi), and TGN46 (trans-Golgi network) (all in red), as described in Materials and Methods. All images are representative of three independent experiments performed and were taken using a 63x objective

To determine the intracellular expression patterns of adenovirally-expressed AFP, DC were transduced for 3 hr and AFP localization was examined by fluorescent microscopy for 24, 48, or 72 hr post-infection. Throughout the observation period, the AFP transgene was detected almost exclusively in the perinuclear space (Fig. 2). Adenovirally-expressed AFP is only transiently present in early endosomes (EEA-1) at 24 h, and not detected in late endosomes/lysosomes (LAMP-1), or the endoplasmic reticulum (KDEL). Some colocalization was observed with ERGIC-53 (ER-Golgi intermediate complex), a compartment which has been implicated in cross presentation [30]. However, the AFP expressed initially in the cytoplasm from the AdV construct colocalizes extensively with Golgi (golgin-97) and *trans*-Golgi (TGN46) compartments, consistent with the routing of secreted proteins through the Golgi apparatus for subsequent release [31].

### Unlike native AFP, adenovirally-encoded eGFP-AFP is retained in the cytoplasm of DC

**Fig. 3:**
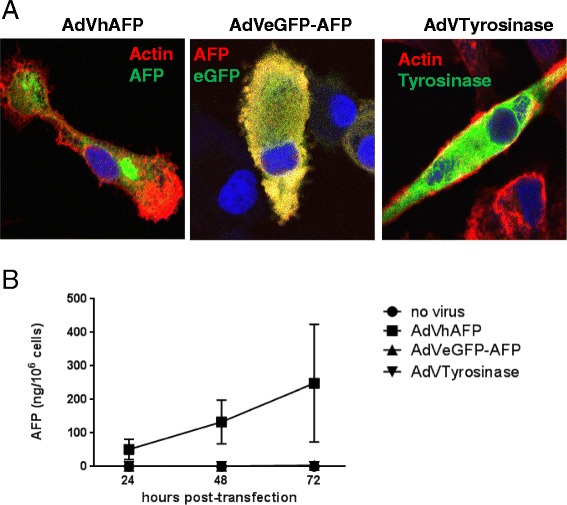
Adenovirally-expressed AFP, but not eGFP-AFP, is secreted by transduced DC. **a** DC were transduced with AdVhAFP (left panel), AdVeGFP-AFP (middle panel), or AdVTyrosinase at MOI 2000 for 3 hr. After 48 hr, cells were fixed and stained for AFP (left and middle panels), actin (left and right panels), or Tyrosinase (right panel). Antibody staining of AdVeGFP-AFP-transduced cells with an anti-AFP antibody reveals that AFP and GFP colocalize, as expected. Representative images from three independent experiments are shown. Images were taken using a 63x objective. **b** DC from healthy donors (n = 3) were transduced as above and cultured in DC media for 24, 48, or 72 hr. Supernatants were analyzed by AFP ELISA. Data are reported as the mean ± SD

Native human AFP expressed by adenovirus (AdVhAFP) is secreted by cells due to a 19 amino acid secretion signal sequence at the N-terminus of AFP [32]. An alternative construct was created in which eGFP was fused to the N-terminus of AFP. Because of the proximity of eGFP to the N-terminus, this alteration masked the signal sequence and resulted in eGFP-AFP accumulation in the cytoplasm of DC. Indeed, while the adenovirally-expressed native AFP localizes primarily to the perinuclear space (Fig. 3a, left panel), the adenovirally-expressed eGFP-AFP fusion protein is detected throughout the cytoplasm (Fig. 3a, middle panel). DC were also transduced with AdVTyrosinase, which encodes the cytoplasmically expressed protein Tyrosinase (Fig. 3a, right panel) for comparison. As anticipated, AdV-driven Tyrosinase and eGFP-AFP displayed similar, cytoplasmically-localized expression patterns.

Clinical laboratory and ELISA assays were employed to determine the concentration of AFP secreted into the supernatants of DC transduced with AdVhAFP or AdVeGFP-AFP. While native AFP is secreted at high levels over three days post-transduction, eGFP-AFP is undetectable in these supernatants (Fig. 3b), further confirming that the addition of eGFP on the AFP N-terminus disrupts and inactivates the secretion signal.

### T cell responses in HD and HCC patients are differentially induced by secreted and cytoplasmically-retained AFP constructs

**Fig. 4:**
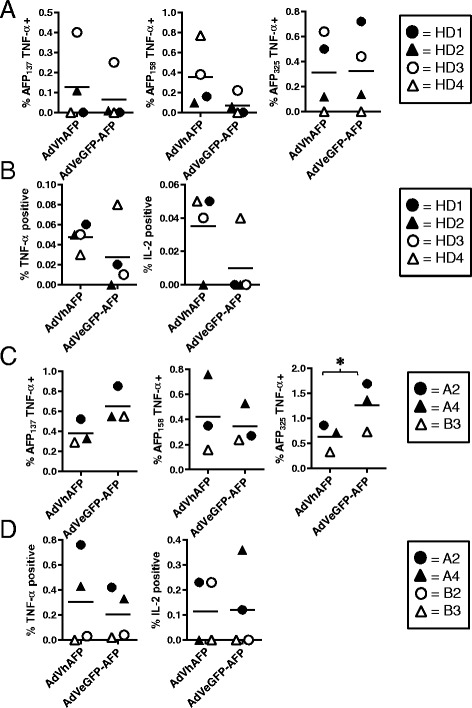
Ability of AdV-transduced DC to induce AFP-specific T cell responses. **a** and (**b**) DC from HLA-A2+ HDs (n = 4) were transduced with AdVhAFP or AdVeGFP-AFP at MOI 2000 for 3 hr, then co-cultured with autologous PBMC for 12–13 days. **a** CD8+ T cells were restimulated with autologous DC (loaded with AFP as in the initial stimulation) for an additional 10d, and then analyzed for AFP-specific intracellular TNF-α production against three immunodominant HLA-A2-restricted peptides. **b** CD4+ T cells were collected after the initial stimulation and analyzed for AFP-specific intracellular cytokine production. (C and D) DC from HLA-A2+ HCC patients (n = 3–4) were transduced with AdVhAFP or AdVeGFP-AFP at MOI 2000 for 3 hr, then co-cultured with autologous PBMC for 12–13 days. **c** CD8+ T cells were collected and analyzed for AFP-specific intracellular TNF-α production against three immunodominant HLA-A2-restricted peptides. **d** CD4+ T cells were collected and analyzed for AFP-specific intracellular cytokine production. For each panel, the solid lines represent the mean value. *, *P* < 0.05

We hypothesized that the presence or absence of the AFP secretion signal would impact the ability of transduced DC to activate AFP-specific T cells. Healthy donor PBMC were stimulated with autologous DC transduced with either AdVhAFP or AdVeGFP-AFP, and CD8+ T cells were analyzed 23 days later for cytokine production upon recognition of three immunodominant, HLA-A2-restricted AFP peptides (Fig. 4a). Four of the 8 tested donors expanded detectable AFP-specific T cells under these short-term stimulation conditions. CD8+ T cell responses against AFP_137_ and AFP_158_ were superior in the AdVhAFP group, whereas both AdVhAFP and AdVeGFP-AFP generated AFP_325_-specific responses, indicating some epitope specific presentation differences between the two constructs. Neither group was statistically significantly superior (global F-test, p = 0.15). We also measured TNF-α and IL-2 production by AFP-specific CD4+ T cells in healthy donors. Again, DC transduced with the secreted AdVhAFP construct generated higher frequency helper responses than the cytoplasmically-retained AdVeGFP-AFP construct (Fig. 4b) although statistical significance was not reached between the groups (TNF p = 0.25, IL-2 p = 0.13).

We next tested the T cell stimulatory activity of these AdVs in PBMC from AFP-positive HCC patients. Four of the 6 HCC PBMC samples expanded detectable AFP-specific T cells. Unlike what we observed in healthy donors, DC transduced with the cytoplasmically-retained AdVeGFP-AFP induced superior or equivalent CD8+ T cell responses (with some epitope-specific differences) versus the AdVhAFP group (Fig. 4c). AFP_137_ differences approached significance (p = 0.06) and AFP_325_ differences were highly significant (p < 0.0001). HCC patient CD4+ T cells were also efficiently activated by both constructs (Fig. 4d), suggesting that in individuals with prior exposure to tumor-derived AFP, but not in healthy donors, robust AFP-specific T cell responses can be induced by both secreted and cytoplasmically-retained AFP.

### AFP protein upregulates maturation markers and downregulates endocytic receptors on DC

**Fig. 5:**
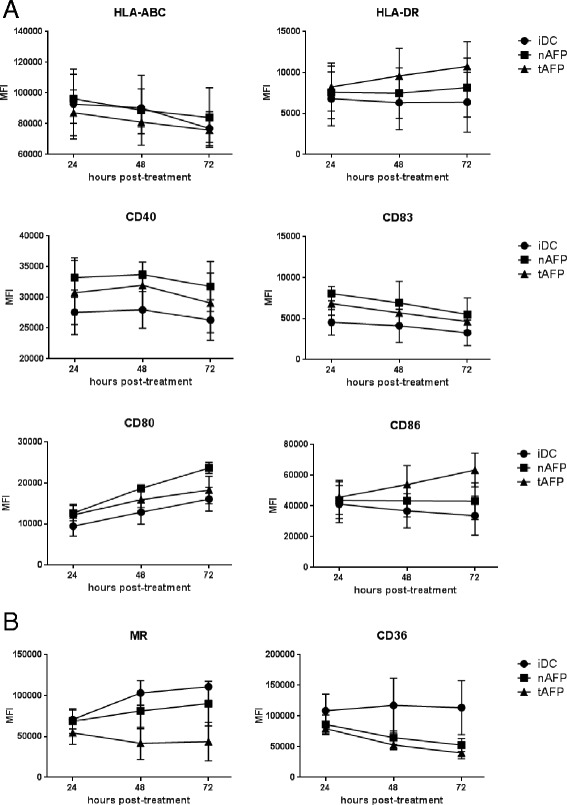
Phenotype of AFP-loaded DC. **a** and (**b**) Immature DC (iDC) from healthy donors (n = 3) were left untreated or cultured with nAFP and tAFP (10 μg/ml) in DC media for 24, 48, or 72 hr. Cells were stained for (**a**) antigen presentation and costimulatory markers and (**b**) endocytic receptors, and analyzed by flow cytometry. Mean fluorescence intensity (MFI) is reported as the mean ± SD

We have recently shown that monocytes cultured with HCC tumor-derived AFP (tAFP), but not normal cord blood-derived AFP (nAFP), fail to fully differentiate into DC, despite the fact that nAFP and tAFP isoforms only differ at one carbohydrate group [33]. Here, we next investigated the effects of nAFP and tAFP on DC that were first differentiated before being exposed to AFP. Compared to untreated iDC, nAFP- and tAFP-treated DC expressed slightly higher levels of several antigen presentation and costimulatory molecules (Fig. 5a). Together with our previous study, these data underscore the multifunctional nature of AFP: potently suppressive in monocytes, yet somewhat stimulatory in previously differentiated DC. Expression of the endocytic receptors MR and CD36, however, was diminished in nAFP- and tAFP-treated DC (Fig. 5b), suggesting that AFP protein negatively regulates the endocytic activity of DC.

### Intracellular trafficking of endocytosed AFP

**Fig. 6:**
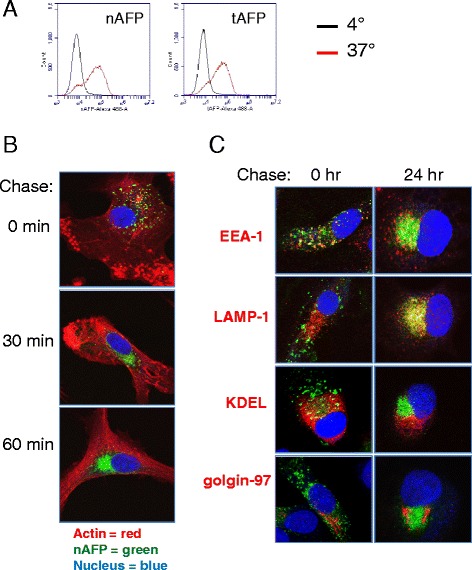
Intracellular trafficking of endocytosed AFP. **a** nAFP and tAFP were Alexa Fluor 488-labeled. Human monocyte-derived DC were co-cultured with AFP (10 μg/ml) for 1 hr at 4 °C or 37 °C, and analyzed by flow cytometry. **b** DC were co-cultured with fluorescently-labeled nAFP for 2 hr, then chased with media alone for the indicated times. Cells were then fixed, stained for actin (described in Materials and Methods), and analyzed by confocal microscopy. **c** DC were incubated with fluorescently-labeled nAFP for 2 hr, then chased with media alone for the indicated times. Cells were then fixed and stained for EEA-1 (early endosomes), LAMP-1 (late endosomes/lysosomes), KDEL (endoplasmic reticulum/ER), and golgin-97 (Golgi) (all in red). All images are representative of three independent experiments performed and were taken using a 63x objective

To understand the dynamics of AFP internalization by DC, we first labeled nAFP and tAFP with Alexa Fluor 488 and verified by flow cytometry that both proteins were efficiently taken up by DC (Fig. 6a). AFP endocytosis was further examined by fluorescent microscopy. After a one hour pulse of DC with fluorescently-labeled nAFP, cells were chased with media alone for 0, 30, and 60 min (Fig. 6b). Immediately following the AFP antigen pulse, endocytic pockets containing nAFP are observed throughout the cytoplasm. This was further examined with live cell imaging which shows that AFP is detectable within DC within 30 s (data not shown). By 30 min post-pulse, nAFP exhibits a perinuclear localization. Similar trafficking patterns were observed with tAFP (*data not shown*). A recent report [30] identified a perinuclear endosomal recycling compartment (ERC) in DC which contain high levels of MHC class I molecules, and which played a role in cross-presentation. We stained our AFP loaded DC for MHC class I and did not see colocalization (not shown), indicating that the peri-nuclear AFP is not in an MHC-loaded ERC. Colocalization of nAFP in intracellular compartments was examined after a one hour pulse and 0 or 24 h chase (Fig. 6c). Immediately following the pulse, nAFP colocalized with early endosomes (EEA-1), but not late endosomes/lysosomes (LAMP-1), endoplasmic reticulum (KDEL), or Golgi (golgin-97). At 24 h post-pulse, nAFP exhibited extensive colocalization with LAMP-1, consistent with a classical MHC Class II processing/presentation paradigm. Similar trafficking patterns were observed with tAFP (*data not shown*).

### DC endocytose AFP primarily via the mannose receptor

**Fig. 7:**
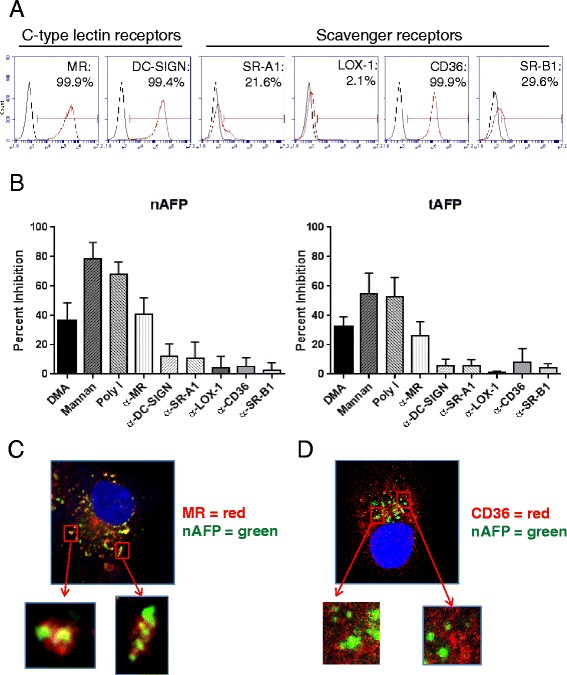
DC endocytose AFP primarily via the mannose receptor. **a** DC were stained for flow cytometric analysis. Red histogram represents endocytic receptor staining; black histogram represents isotype control staining. Data from one representative healthy donor (of three total HD tested) is shown. **b** DC were pre-treated with inhibitors for 30 min, and co-cultured with fluorescently-labeled nAFP (top panels) or tAFP (bottom panels) for an additional 1 hr. Percent inhibition was calculated based on untreated control cells. Columns, mean of four HD; bars, standard deviation. **c** and (**d**) DC were incubated with fluorescently-labeled nAFP for 2 hr, then fixed and stained for MR (**c**) or CD36 (**d**). All images are representative of three independent experiments performed and were taken using a 63x objective

Although an AFP cell surface receptor has been partially characterized biochemically [17], it has not been definitively cloned. Several molecules have been proposed as putative receptors for AFP, including the mannose receptor (MR/CD206) and several scavenger receptors (SR-A1, LOX-1, CD36, SR-B1) [34]. We first examined DC for expression of these receptors. As depicted in Fig. 7a, DC express high levels of MR, DC-SIGN, and CD36. To identify the mechanism(s) of AFP uptake, DC were pretreated with inhibitors against pinocytosis (DMA), CLR-mediated endocytosis (mannan), and scavenger receptor-mediated endocytosis (polyinosinic acid; poly I). Cells were then pulsed with fluorescently-labeled nAFP and tAFP and uptake inhibition calculated (Fig. 7b). While the blockade of receptor-independent pinocytosis by DMA displayed moderate suppressive activity, uptake of nAFP and tAFP was substantially reduced by mannan and poly I, indicating that CLR- and scavenger receptor-mediated endocytosis accounts for the majority of AFP protein internalization. AFP internalization by DC was not abrogated by GalNAc, which inhibits galactose-specific CLRs (i.e. MGL) (*data not shown*). We also targeted individual CLRs and scavenger receptors using blocking antibodies. Of those tested, only the MR blocking antibody demonstrated suppressive activity, suggesting that the primary mechanism of AFP uptake in DC is through MR-mediated endocytosis.

We further analyzed the role of MR in AFP uptake using fluorescent microscopy. Immediately after DC were pulsed with fluorescently-labeled nAFP, endocytic pockets containing MR and nAFP, together and co-localized, were observed throughout the cytoplasm of DC, further supporting the role of MR in AFP endocytosis (Fig. 7c). Similar trafficking patterns and MR co-localization were observed with tAFP (*data not shown*). No colocalization was observed, however, between endocytosed nAFP and the scavenger receptor CD36 (Fig. 7d).

Because HCC cells are also capable of taking up AFP [35], endocytosis of nAFP and tAFP by the HCC cell line HepG2 was also assessed. HepG2 cells take up comparable levels of both nAFP and tAFP (Additional file 1: Figure S1A), although to a lesser degree than DC (Fig. 6a). We also confirmed that AFP is internalized by HepG2 cells and not simply bound to the cell surface (Additional file 1: Figure S1B). Unlike DC, HepG2 cells do not express CLRs, but express high levels of CD36 and moderate levels of LOX-1 and SR-B1 (Additional file 1: Figure S1C). By utilizing the same uptake inhibition strategy, we demonstrate that AFP uptake occurs primarily through pinocytosis and scavenger receptors, in contrast to DC (Additional file 1: Figure S1D).

### AFP-specific T cell responses induced by protein-loaded DC occur in both MR-dependent and –independent manners

**Fig. 8:**
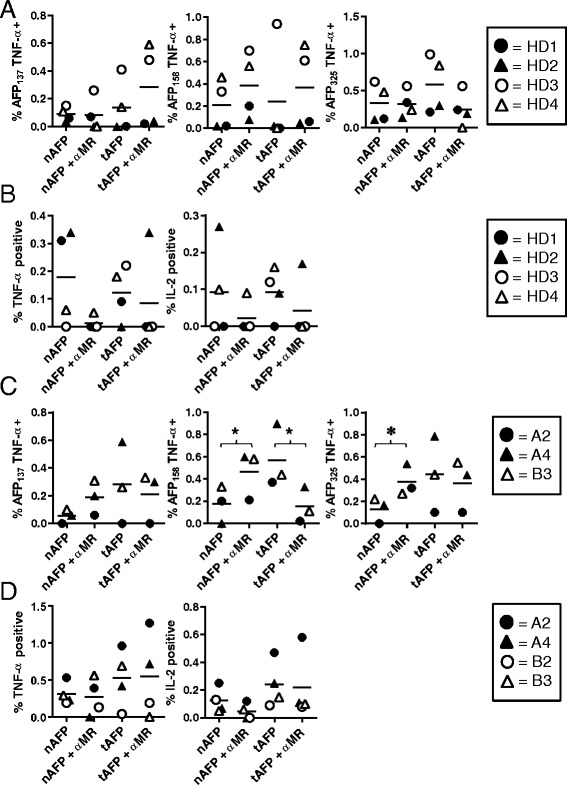
Ability of AFP protein-loaded DC to induce AFP-specific T cell responses. **a** and (**b**) DC from HLA-A2+ HDs (n = 4) were cultured with nAFP or tAFP (at 10 μg/ml for 2 hr) with or without pre-treatment with MR blocking antibody (at 10 μg/ml for 30 min), matured for an additional 24 h, then co-cultured with autologous PBMC for 12–13 days. **a** CD8+ T cells were restimulated with autologous DC (loaded with AFP as in the initial stimulation) for an additional 10d, and then analyzed for AFP-specific intracellular TNF-α production against three immunodominant HLA-A2-restricted peptides. **b** CD4+ T cells were collected after the initial stimulation and analyzed for AFP-specific intracellular cytokine production. **c** and (**d**) DC from HLA-A2+ HCC patients (n = 3–4) were cultured with nAFP or tAFP (at 10 μg/ml for 2 hr) with or without pre-treatment with MR blocking antibody (at 10 μg/ml for 30 min), matured for an additional 24 h, then co-cultured with autologous PBMC for 12–13 days. (C) CD8+ T cells were collected and analyzed for AFP-specific intracellular TNF-α production against three immunodominant HLA-A2-restricted peptides. (D) CD4+ T cells were collected and analyzed for AFP-specific intracellular cytokine production. For each panel, the solid lines represent the mean value. *, *P* < 0.05

Previous studies by our group have shown AFP protein-loaded immature DC to be inferior to AdVhAFP-transduced DC at inducing AFP-specific CD4+ and CD8+ T cell responses [36, 37]. In this study, the AFP protein-loaded DC were matured with LPS/IFN-γ prior to T cell stimulation to determine if a more optimized protein loading procedure would activate strong T cell responses. We also wanted to assess both glycoforms of AFP (nAFP and tAFP), as well as the significance of MR-mediated AFP endocytosis in the activation of T cells by DC. Healthy donor DC were pulsed with nAFP or tAFP in the presence or absence of MR blocking antibody, matured for an additional 24 h, then co-cultured with autologous PBMC. Several days later, CD8+ T cells were analyzed for cytokine production in response to AFP-derived peptides (Fig. 8a). This allowed us to detect cross-presentation of the exogenously added protein, compared to the cytoplasmically delivered virally encoded AFP tested above (Fig. 4). In general, multi-epitope CD8^+^ T cell responses were generated from both nAFP or tAFP protein, which was not inhibited by MR blockade, indicating that the glycosylation state and route of uptake of AFP by DC are not critical parameters for priming CD8^+^ T cells of HD. The frequencies of CD8^+^ T cells expanded from protein cross-presentation were generally similar to that of cytoplasmic antigen in the matured DC although the exogenous protein loaded DC activated significantly more AFP_325_ or AFP_158_-specific T cells (p = 0.007). Robust HD CD4^+^ T cell responses, however, were almost completely abrogated by MR blockade in 3 of 4 donors (Fig. 8b), suggesting that MR-mediated routing of internalized AFP into the MHC Class II pathway is important for AFP-specific CD4^+^ T cell priming. Exogenous protein yielded higher frequencies of CD4^+^ T cell responses than either virally encoded form.

Protein-loaded HCC patient DC were next evaluated for their T cell stimulatory activity. As with healthy donors, MR blockade did not compromise the ability of these DC to cross-present antigen and induce high frequency CD8^+^ T cell activation (Fig. 8c). In fact, MR blockade significantly increased the frequency of AFP_158_ and AFP_325_-specific T cells stimulated by nAFP loaded DC (p = 0.02 and 0.046, respectively). While inhibiting tAFP stimulation of AFP_158_-specific CD8^+^ T cells (p = 0.0008). Importantly, MR blockade had a negligible effect on AFP-specific CD4^+^ T cell activation in HCC patients (Fig. 8d). This is in contrast to the reduced CD4^+^ T cell responses with MR blockade in healthy donors. These data suggest that HCC patients with AFP-secreting tumors may have been previously primed by circulating tAFP, and that our *in vitro* cultures are testing the loaded DC ability to boost these T cells. Indeed, the frequencies of AFP-specific CD4+ T cells are much higher (Figs. 4d and 8d) than from healthy donors (Figs. 4b and 8b). Together with our AdV-delivered antigen results (Fig. 4), these data show that T cells from AFP-naïve healthy donors and AFP-experienced HCC patients are differentially activated by alternative strategies and pathways of DC antigen loading.

## Conclusions

Numerous cell surface receptors on DC, including MR, DEC-205, DC-SIGN, and several scavenger receptors, mediate the endocytosis of exogenous antigens. Internalized antigen is generally shuttled into lysosomes, where it is subsequently degraded and presented in the context of MHC Class II molecules. Alternatively, DC are capable of re-routing internalized antigen into the MHC Class I pathway via several potential mechanisms [38]. This process, known as cross-presentation, is essential for the development of protective anti-tumor CD8+ T cell immunity. Importantly, recent evidence suggests that the divergence of internalized antigen into either MHC Class I or Class II pathways may be dictated by the particular cell surface receptor that was responsible for endocytosis of the antigen. We have recently shown that AFP, a secreted oncofetal antigen that is over-expressed by more than half of HCC tumors, is efficiently internalized by human monocyte-derived DC. Here, to identify the endocytosis mechanism(s) responsible for AFP internalization and to determine the downstream impact on T cell responses, we co-cultured DC with fluorescently-labeled AFP in the presence or absence of inhibitors of specific endocytic pathways. Pretreatment of DC with mannan, an MR inhibitor that competitively blocks endocytosis of mannose-rich structures, abrogated AFP uptake, while similar treatment with polyinosinic acid, a specific inhibitor of scavenger receptors, blocked between 50-70 % of AFP internalization. These data are consistent with the observed high levels of MR and the scavenger receptors SR-A1 and CD36 expressed by the DC. MR is also known to be expressed on circulating human BDCA1^+^ DC [39]. Because AFP is also taken up by HCC cells, where it is thought to regulate apoptosis and steroid receptor-mediated cell growth, we performed similar endocytosis blockade experiments using the HCC cell line HepG2. In contrast to DC, uptake of AFP by HepG2 cells was mediated by pinocytosis and several scavenger receptors, including LOX-1 and CD36.

Vaccines, including cancer vaccines, have utilized many approaches to convey antigen to stimulatory antigen presenting cells. *Ex vivo* DC loading strategies include tumor fusion, tumor lysate loading (with a variety of tumor killing strategies), purified protein loading, antibody conjugate targeting, long peptide loading, and MHC-restricted short peptide epitope pulsing [40]. Each of these approaches has been used in clinical trials with variable immunologic and therapeutic outcomes [41–43]. The role of the antigen uptake pathway in not generally addressed in these studies.

The MR has been shown to be highly expressed on cultured DC and to be a very efficient antigen uptake receptor [44]. To improve the uptake efficiency of some antigens, conjugation of mannose groups has been tested and shown to improve uptake by DC [45]. This CD206 pathway allows DC to accumulate a large amount of a variety of antigen molecules as well as microbes with mannosylated glycoproteins very quickly [3], with very efficient receptor recycling. Depending on the antigen glycosylation state, as little as 15 min of antigen uptake can result in maximal T cell activation [44]. Here, we show that uptake of AFP is extremely efficient, with intracellular antigen detected immediately after pulsing. The mature, circulating form of AFP contains approximately 4 % carbohydrates by weight, including N-acetyl-glucosamine (1.2 %), mannose (2.2 %), sialic acid (0.9 %) and small amounts of glucose [46]. Binding of AFP regions to MR were predicted in silico by an algorithm designed by one of us [47], with AFP 485–493 and AFP 492–500 in domain 3 being critical. As we [33] and others [48–53] have shown, AFP can have immune suppressive effects on DC and we have shown that a significant amount of that activity is due to low molecular mass (LMM) co-purifying molecules bound to AFP that is internalized with the AFP. Here, we have shown that MR-mediated uptake of both nAFP and tAFP is very efficient, allowing for a quick accumulation of AFP protein in early endosomes, which can be detected in the perinuclear space for at least 72 h. This uptake pathway may, therefore, enhance the ability of the co-purifying molecules (known to include fatty acids, bilirubin and neopterin [54, 55]) to be concentrated inside DC. Blockade of CD206 reduced HD CD4+ T cell responses, suggesting that scavenger receptor-mediated uptake does not support efficient antigen routing for MHC class II presentation, while CD206 mediated routing through early endosomes is the most efficient route. Unexpectedly, tAFP and nAFP behave similarly as antigen sources for CD8^+^ and CD4^+^ T cell activation. The only significant difference we detected was that MR blockade improved CD8^+^ T cell responses in HCC patients from nAFP loaded DC. The frequencies of tAFP-induced T cells was also higher frequency in HCC patients than for nAFP.

Replication-deficient AdVs are also an established gene delivery vehicle, capable of transducing both dividing and non-dividing cells and inducing prolonged transgene expression (up to 14 days) into transduced cells [56]. Type 5 AdV bind to the cell surface Coxsackie-adenovirus receptor (CAR) and cell-surface integrins, leading to clathrin-mediated endocytosis. The virus-containing macropinosomes are lysed, releasing the contents into the cytosol [57]. We found that the adenovirally-delivered cytoplasmic eGFP-AFP was diffusely localized throughout the cell, without any accumulation in the perinuclear regions. The superiority of the native secreted form of AFP for priming of both CD4+ and CD8+ T cell responses in HD may relate to the high level accumulation of antigen over several days throughout the ERGIC, Golgi and TGN. Our observations are also similar to those described by Fukasawa *et al*., in which recombinant native AFP was routed into the secretory pathway of various cell lines via the Golgi, whereas an AFP construct lacking the N-terminal secretion signal was retained in the cytoplasm and failed to be secreted [58].

We hypothesize that healthy donor T cells have been exposed to nAFP, taken up via CD206 and SR pathways, and presented to CD4+ and CD8+ T cells, during fetal development through birth. This is in contrast to other types of self antigens, like cytoplasmically expressed proteins from adult tissues like melanocyte lineage antigens. HCC patients with tAFP-expressing tumors have been further exposed as adults to circulating tAFP. While we demonstrate here that both nAFP and tAFP are efficiently endocytosed by DC via CD206 and SRs, we recently demonstrated that tAFP binds to potently immune suppressive molecules that can significantly impair DC function and T cell stimulatory activity [33].

Hepatocellular carcinoma (HCC), the most common form of liver cancer, is the second leading cause of cancer-related death worldwide [59]. Current therapies for advanced HCC are marginally effective and can exacerbate underlying liver disease. The ability of immunotherapy to elicit nontoxic, systemic, long-lived anti-tumor activity therefore makes it an attractive area of investigation, and there have been a few intriguing trials with improved outcomes for vaccinated HCC patients [60, 61]. Importantly, a minority of HCC tumors are spontaneously infiltrated by lymphocytes [62], and to date, HCC has not been one of the more responsive tumor types with checkpoint blockade with anti-CTLA-4 or anti-PD-1 [63]. It may be critical to first drive a tumor antigen immune response with vaccination to create a setting for response to other therapies.

## Methods

### Reagents

Human cord serum nAFP (Cell Sciences; purity >95 % by SDS-PAGE), and HCC cell line culture-derived tAFP (Bio-Rad; purity >95 % by SDS-PAGE) were added to cultures at 10 μg/ml. Alexa Fluor 488-conjugation of nAFP and tAFP was performed using an Alexa Fluor 488 protein labeling kit (Life Technologies). Overlapping peptides (20-mer amino acids overlapping by 10) spanning the AFP protein were from Mimotopes (>80 % purity). Lyophilized peptides were reconstituted in DMSO and pooled. AFP-derived peptides (AFP_137–145_: PLFQVPEPV; AFP_158–166_: FMNKFIYEI; AFP_325–334_: GLSPNLNRFL) were purchased from the University of Pittsburgh Peptide Synthesis Facility. E1- and E3-deleted adenoviruses encoding human AFP (AdVhAFP) and enhanced green fluorescent protein (eGFP)-tagged AFP (AdVeGFP-AFP) were acquired from BDP/NCI-Frederick and the University of Pittsburgh Vector Core, respectively. AdVTyrosinase was also acquired from the University of Pittsburgh Vector Core. Viral titers were tested using the Adeno-X Rapid Titer Kit (Clontech Laboratories) and multiplicity of infection (MOI) used to infect DC was based on the values obtained.

### Cell lines

HepG2 hepatoma (ATCC #HB-8065) and T2 (HLA-A2+; ATCC #CRL-1992) cell lines were cultured in RPMI 1640 medium, supplemented with 10 % fetal bovine serum, 1 % penicillin-streptomycin, and 1 % L-glutamine (all reagents from Life Technologies). Cultures were maintained in a humidified 37 °C incubator under 5 % CO_2_ tension.

### Isolation of PBMCs

Peripheral blood mononuclear cells (PBMC) were obtained from 8 healthy donors (HD) and from 6 HCC patients banked from an AFP peptide-pulsed DC vaccine clinical trial [22] (UPCI #04-001; UCLA IRB #00-01-026, IND BB9395; informed consent was obtained from all patients and donors). Limited patient data are listed in Additional file 2: Table S1. PBMC were separated from blood using gradient centrifugation (Ficoll-Paque, GE Healthcare).

### DC preparation

CD14+ monocytes were isolated from PBMC using magnetic cell sorting (Miltenyi Biotec) and cultured for 5 days in 800 IU/ml rGM-CSF (Sargramostim; Genzyme) and 500 IU/ml rIL-4 (eBioscience; purity >98 % by SDS-PAGE). Where noted, DC were matured with IFN-γ (1000 IU/ml; PeproTech) and lipopolysaccharide (LPS, 250 ng/ml; Sigma-Aldrich) for an additional 24 h prior to collection. DC were transduced with AdVhAFP, AdVeGFP-AFP, or AdVTyrosinase for 3 hr in serum-free media at MOI 2000.

### Flow cytometry

Endocytic receptors were stained using the following antibodies: CD206/MR (eBioscience), DC-SIGN, CD36 (both from BD Biosciences), SR-A1 (R&D Systems), LOX-1 (BioLegend), and SR-B1 (Novus Biologicals). DC and T cell phenotypes were examined using antibodies against the following markers: HLA-ABC (BioLegend), CD206, CD40, CD80, CD83, IL-2 (BD Biosciences), CD4, CD8, TNF-α, IL-2, and HLA-DR (Beckman Coulter). Data were acquired with an Accuri C6 cytometer (BD Biosciences) and analyzed using CFlow Plus software.

### Uptake inhibition assays

DC or HepG2 cells were pretreated with inhibitors in serum-free media for 30 min at 37 °C. Without washing, fluorescently-labeled protein was added at 10 μg/ml for 1 hr at 37 °C. After washing, MFI of internalized protein was quantitated by flow cytometry. Percent inhibition was calculated as: [(MFI of untreated cells) – (MFI of treated cells)]/(MFI of untreated cells) × 100 %. The following blocking antibodies were used: CD206 (clone 15–2; BioLegend), DC-SIGN (clone 120507), SR-A1 (clone 351620), LOX-1 (clone 331212; all from R&D Systems), CD36 (clone 185-1G2; NeoMarkers), and SR-B1 (rabbit polyclonal; Novus Biologicals). Dimethylamiloride (DMA; 100 μM), mannan (300 μg/ml), and polyinosinic acid (Poly I; 50 μg/ml) were purchased from Sigma.

### Confocal immunofluorescence staining and imaging

DC cultured in 8-well chamber slides (Nunc) were fixed (4 % paraformaldehyde), permeabilized (0.1 % Triton X-100), and stained with rhodamine phalloidin (Life Technologies) and DRAQ5 (eBioscience), to label F-actin and nuclei, respectively. The following primary antibodies were used for staining cells: AFP (AbD Serotec), CD206 (BioLegend), CD36, Tyrosinase, EEA-1, LAMP-1, KDEL, ERGIC-53 (all Santa Cruz Biotechnology), golgin-97 (Life Technologies), TGN46 (Sigma), mouse and rabbit IgG isotype controls (R&D Systems), and anti-Alexa Fluor 488 (to amplify the fluorescent signal of Alexa Fluor 488-labeled protein; Life Technologies). The following secondary antibodies were used: goat anti-mouse Alexa Fluor 488 and 555, goat anti-rabbit Alexa Fluor 488 and 555 (all from Cell Signaling Technology), donkey anti-goat Cy3 (Jackson ImmunoResearch), donkey anti-mouse Alexa Fluor-488, and donkey anti-rabbit Alexa Fluor-488 (both Life Technologies). Images were acquired using a Leica TCS-SL confocal microscope.

### Quantification of secreted AFP by ELISA

Secretion of AFP and eGFP-AFP by adenovirus vector-transfected DCs was quantified using DuoSet ELISA development system for human AFP (R&D Systems) per the manufacturer’s protocol.

### T cell stimulation and cytokine production assays

AFP-loaded DC (via AdVhAFP transduction or AFP protein pulse) from HLA-A2+ HD or HCC patients were co-cultured with autologous monocyte-depleted PBMC at a 1:10 ratio (DC: PBMC) in RPMI 1640 plus 10 % human AB serum plus pen/strep/L-G In plus 20 IU/ml IL-2 and 5 ng/ml IL-7. Cultures were supplemented with fresh media containing IL-2 and IL-7 every 3–4 days. After 12–13 days, HD CD4+ T cells and HCC patient CD8+ and CD4+ T cells were analyzed for AFP-specific cytokine production, while HD CD8+ T cells were isolated by magnetic bead selection (Miltenyi Biotec) and restimulated with autologous DC (loaded with AFP as in the initial stimulation) for an additional 10d, and then analyzed for AFP-specific cytokine production. For CD8+ T cell intracellular cytokine staining, cells were stimulated in the presence of brefeldin A with T2 cells pre-loaded with one of three immunodominant AFP-derived peptides (AFP_137–145_, AFP_158–166_, and AFP_325–334_; all at 10 μg/ml). Six hours later, T cells were stained for CD8, fixed, permeabilized, and stained for intracellular TNF-α. For CD4+ T cell intracellular cytokine staining, cells were stimulated in the presence of brefeldin A with autologous DC pre-loaded with the AFP peptide pool (at 60 μg/ml). Six hours later, T cells were stained for CD4, fixed, permeabilized, and stained for intracellular IL-2 and TNF-α.

### Statistical analysis

To determine the statistical significance of differences in AFP antigen form and uptake pathways, Figs. 4 and 8 were analyzed as follows. Mixed effects ANOVA were fit to the data, and the distributions of the residuals were visually checked using Q-Q plots. While some of the data demonstrated serious departures from the distributional assumption, these departures were not present in any of the analyses with statistically significant results. *P* values < 0.05 are considered significant and noted with asterisks in the figures.

## Additional files

Additional file 1: Figure S1. HepG2 cells endocytose AFP primarily via pinocytosis and scavenger receptors. (A) nAFP and tAFP were Alexa Fluor 488-labeled. HepG2 cells were co-cultured with AFP (10 μg/ml) for 1 hr at 4 °C or 37 °C, and analyzed by flow cytometry. (B) HepG2 cells were co-cultured with fluorescently-labeled nAFP for 2 hr, then fixed, stained for actin (described in Materials and Methods), and analyzed by confocal microscopy. (C) HepG2 cells were stained for flow cytometric analysis. Red histogram represents endocytic receptor staining; black histogram represents isotype control staining. Data from one representative experiment (of three total) is shown. (D) HepG2 cells were pre-treated with inhibitors for 30 min, and co-cultured with fluorescently-labeled nAFP (left panel) or tAFP (right panel) for an additional 1 hr. Percent inhibition was calculated based on untreated control cells. Columns, mean of three independent experiments; bars, standard deviation. Additional file 2: Table S1. HCC patient demographics.
