# Supplementary material for: Route of antigen delivery impacts the immunostimulatory activity of dendritic cell-based vaccines for hepatocellular carcinoma
Source: J Immunother Cancer. 2015 Jul 21;3:32. doi: 10.1186/s40425-015-0077-x (PMC4509479; doi:10.1186/s40425-015-0077-x)
Supplement: Additional file 1: Figure S1. — HepG2 cells endocytose AFP primarily via pinocytosis and scavenger receptors. (A) nAFP and tAFP were Alexa Fluor 488-labeled. HepG2 cells were co-cultured with AFP (10 μg/ml) for 1 hr at 4 °C or 37 °C, and analyzed by flow cytometry. (B) HepG2 cells were co-cultured with fluorescently-labeled nAFP for 2 hr, then fixed, stained for actin (described in Materials and Methods), and analyzed by confocal microscopy. (C) HepG2 cells were stained for flow cytometric analysis. Red histogram represents endocytic receptor staining; black histogram represents isotype control staining. Data from one representative experiment (of three total) is shown. (D) HepG2 cells were pre-treated with inhibitors for 30 min, and co-cultured with fluorescently-labeled nAFP (left panel) or tAFP (right panel) for an additional 1 hr. Percent inhibition was calculated based on untreated control cells. Columns, mean of three independent experiments; bars, standard deviation. [file 40425_2015_77_MOESM1_ESM.pptx]

## Slide 1
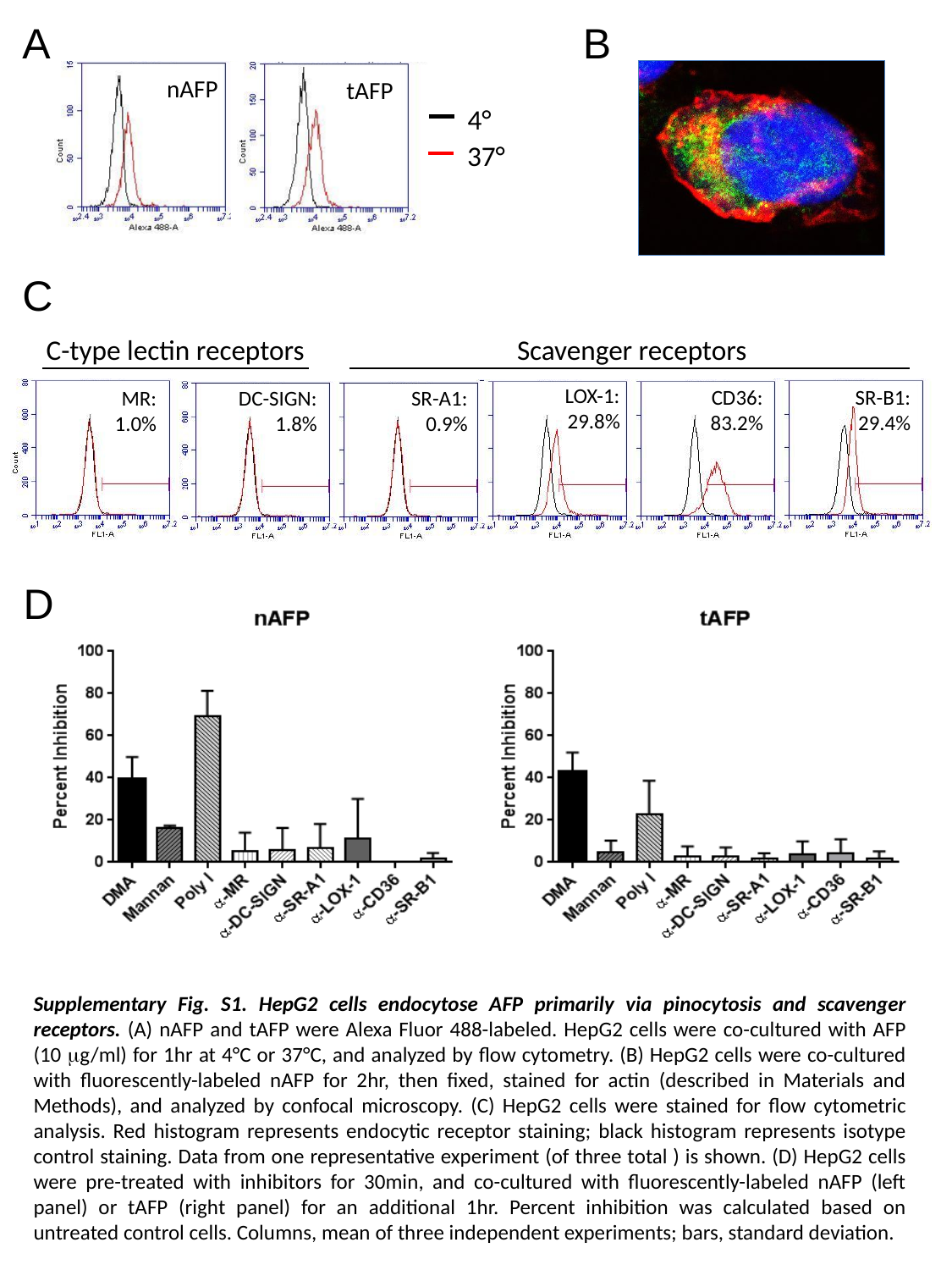

A
B
nAFP
tAFP
4°
37°
C
C-type lectin receptors
Scavenger receptors
LOX-1:
29.8%
SR-B1:
29.4%
CD36:
83.2%
MR:
1.0%
DC-SIGN:
1.8%
SR-A1:
0.9%
D
Supplementary Fig. S1. HepG2 cells endocytose AFP primarily via pinocytosis and scavenger receptors. (A) nAFP and tAFP were Alexa Fluor 488-labeled. HepG2 cells were co-cultured with AFP (10 μg/ml) for 1hr at 4°C or 37°C, and analyzed by flow cytometry. (B) HepG2 cells were co-cultured with fluorescently-labeled nAFP for 2hr, then fixed, stained for actin (described in Materials and Methods), and analyzed by confocal microscopy. (C) HepG2 cells were stained for flow cytometric analysis. Red histogram represents endocytic receptor staining; black histogram represents isotype control staining. Data from one representative experiment (of three total ) is shown. (D) HepG2 cells were pre-treated with inhibitors for 30min, and co-cultured with fluorescently-labeled nAFP (left panel) or tAFP (right panel) for an additional 1hr. Percent inhibition was calculated based on untreated control cells. Columns, mean of three independent experiments; bars, standard deviation.
