# Supplementary material for: Route of antigen delivery impacts the immunostimulatory activity of dendritic cell-based vaccines for hepatocellular carcinoma
Source: J Immunother Cancer. 2015 Jul 21;3:32. doi: 10.1186/s40425-015-0077-x (PMC4509479; doi:10.1186/s40425-015-0077-x)
Supplement: Additional file 2: Table S1. — HCC patient demographics. [file 40425_2015_77_MOESM2_ESM.pptx]

## Slide 1
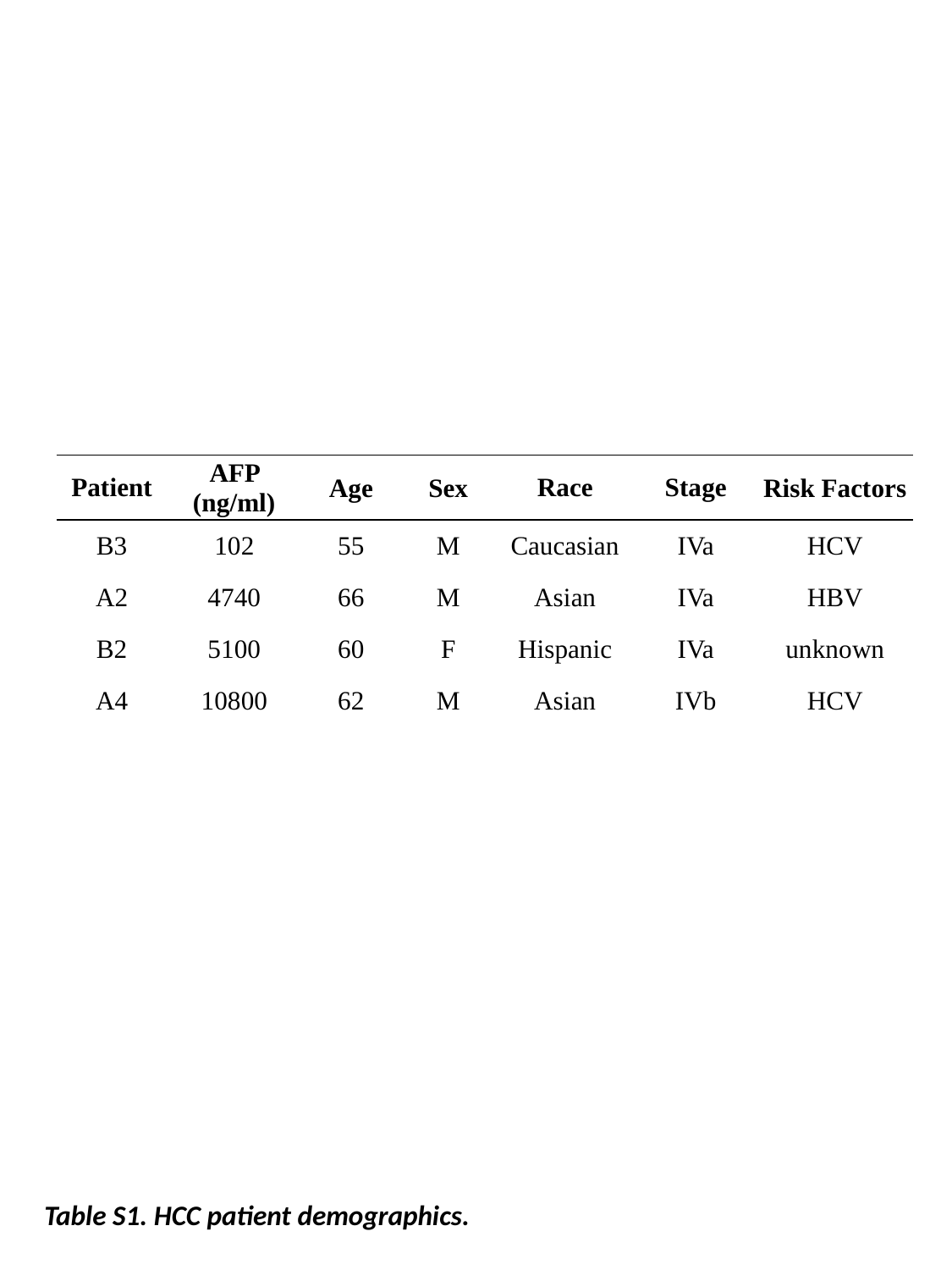

| Patient | AFP (ng/ml) | Age | Sex | Race | Stage | Risk Factors |
| --- | --- | --- | --- | --- | --- | --- |
| B3 | 102 | 55 | M | Caucasian | IVa | HCV |
| A2 | 4740 | 66 | M | Asian | IVa | HBV |
| B2 | 5100 | 60 | F | Hispanic | IVa | unknown |
| A4 | 10800 | 62 | M | Asian | IVb | HCV |
Table S1. HCC patient demographics.
